# Morin Induces Melanogenesis via Activation of MAPK Signaling Pathways in B16F10 Mouse Melanoma Cells

**DOI:** 10.3390/molecules26082150

**Published:** 2021-04-08

**Authors:** SeoYeon Shin, JaeYeon Ko, MinJeong Kim, Nuri Song, KyungMok Park

**Affiliations:** Department of Pharmaceutical Engineering, Dongshin University, 185 gunjae-ro Naju, Jeonnam 58245, Korea; ssy33144@naver.com (S.S.); cp8340@naver.com (J.K.); 6760933@naver.com (M.K.); nuri980424@naver.com (N.S.)

**Keywords:** morin, melanin, tyrosinase, TRP-1/2, MITF, ERK, p38

## Abstract

Morin is a well-known flavonoid, and has been reported to have various properties, such as anti-cell death, antioxidant, and anti-inflammatory properties. Although studies on the biochemical and biological actions of morin have been reported, the melanin biosynthesis effects and molecular mechanisms are unknown. In this study, we first found that morin has the effect of enhancing melanin biosynthesis in B16F10 mouse melanoma cells, and analyzed the molecular mechanism. In this study, we examined the effects of morin on the melanin contents and tyrosinase activity, as well as the protein expression levels of the melanogenic enzymes TRP-1, TRP-2, and microphtalmia-associated transcription factor (MITF) in B16F10 mouse melanoma cells. Morin showed no cytotoxicity in the concentration range of 5–100 μM, and significantly increased the intracellular tyrosinase activity and melanin contents. In mechanism analysis, morin increased the protein expression of TRP-1, TRP-2, and MITF associated with melanogenesis. Furthermore, morin increased phosphorylated ERK and p38 at the early time, and decreased phosphorylated ERK after 12 h. The results suggest that morin enhances melanin synthesis through the MAPK signaling pathways in B16F10 mouse melanoma cells.

## 1. Introduction

Vitiligo is a skin depigmentation disorder. It has been reported that 0.5–1% of the world’s population has this disease, regardless of race or gender [1,2,3]. This skin disease occurs when cutaneous melanocytes are destroyed or non-functional [4,5]. Melanin is produced by melanosomes in the melanocytes, and is scattered in the basal layer of the epidermis. Melanin is an essential component of skin pigmentation, and plays an important role in preventing UV damage [6].

Melanogenesis is regulated by melanogenic enzymes, such as tyrosinase (TYR), tyrosinase-related protein 1 (TRP-1), and tyrosinase-related protein 2 (TRP-2) [7]. Tyrosinase plays a pivotal role in melanogenesis by the hydroxylation of tyrosine into dihydroxyphenylalanine (DOPA), followed by further oxidation of DOPA to DOPA quinone. TRP-2 acts as a dopachrome tautomerase, and catalyzes the rearrangement of dopachrome to form 5,6-dihydroxyindole-2-carboxylic acid (DHICA), and TRP-1 oxidizes DHICA to produce carboxylate indolequinone [8,9,10,11].

Microphthalmia-associated transcription factor (MITF) is a major regulator of the transcription of genes involved in melanin synthesis. Several signaling pathways mediate melanogenesis [12,13]. The p38 mitogen-activated protein kinase (p38 MAPK) and c-Jun N-terminal kinase mitogen-activated protein kinase (JNK MAPK) pathway may upregulate melanogenesis by increasing MITF expression. The extracellular signal-regulated kinase mitogen-activated protein kinase (ERK MAPK)-dependent MITF expression pathway is also involved in melanogenesis [14,15].

Morin (2′,3,4′,5,7-pentahydroxyflavone) is a well-known flavonoid found naturally in various fruits, mulberries, almonds, red wine, and many Chinese herbs [16,17,18]. Previous studies have reported that morin has anti-cell death, antioxidant, and anti-inflammatory properties [19,20,21]. The biochemical and biological actions of morin have been studied, but the effects of melanin synthesis and its mechanism of action are unknown. Therefore, this study focused on analyzing the effect of morin on melanin biosynthesis and its molecular mechanism in B16F10 mouse melanoma cells.

## 2. Results

### 2.1. Effects of Morin on Cell Viability

**Figure 1:**
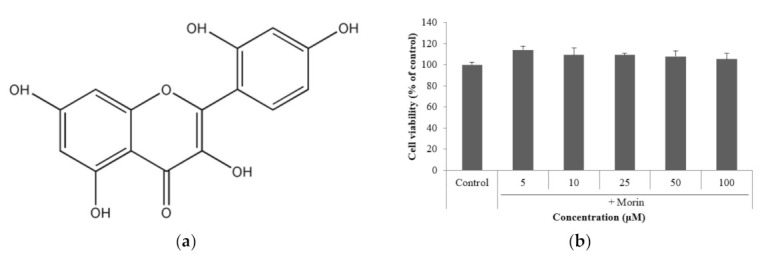
(**a**) Chemical structure of morin. (**b**) Effect of morin on the viability of B16F10 mouse melanoma cells. Cells were treated with morin (5, 10, 25, 50, and 100 μM) for 48 h. The data are presented as the mean ± SD of at least three independent experiments.

To assess the cytotoxicity of morin (Figure 1a), cell viability was measured by MTT assay in B16F10 mouse melanoma cells. Morin was incubated at various concentrations (5–100 μM) for 48 h at 37 °C in a 5% CO_2_ incubator. It did not affect cell viability at any concentration compared to the control (Figure 1b).

### 2.2. Effects of Morin on Melanin Contents

**Figure 2:**
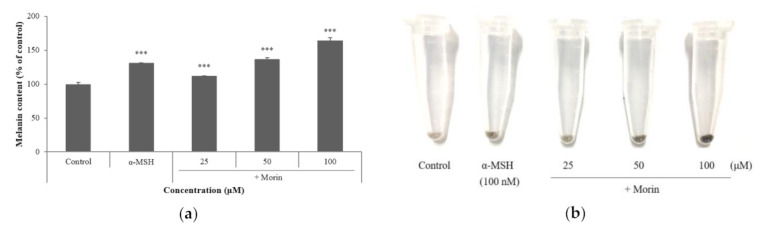
(**a**) Effect of morin on melanin contents. B16F10 mouse melanoma cells were treated with morin (25, 50, and 100 μM) for 72 h. (**b**) Cell precipitation following centrifugation is indicated; α-MSH (100 nM) was used as the positive control. The data are presented as the mean ± SD of at least three independent experiments; *** *p* < 0.001 compared with the control.

Cytotoxicity and melanin content measurements in B16F10 mouse melanoma cells were performed at 25–100 μM of morin. Melanin content increased in a concentration-dependent manner (Figure 2a). Morin showed a higher melanin content at 50 μM than the positive control α-MSH (100 nM). In addition, the color change was visually confirmed in the pellets of the treated groups (Figure 2b). Morin showed a darker pellet than that of a-MSH at 50 μM.

### 2.3. Effects of Morin on Mushroom and Intracellular Tyrosinase Activity

**Figure 3:**
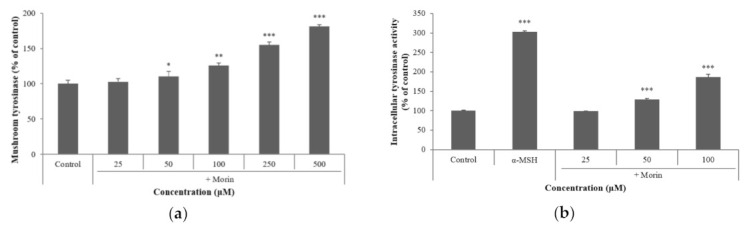
Effect of morin on tyrosinase activity. (**a**) The effects of morin on mushroom tyrosinase activity were determined as described in the Materials and Methods section. (**b**) B16F10 mouse melanoma cells were treated with morin (25, 50, and 100 μM) for 72 h, and the intracellular tyrosinase activity was determined as described in the Materials and Methods section; α-MSH (100 nM) was used as the positive control. The data are presented as the mean ± SD of at least three independent experiments; * *p* < 0.05, ** *p* < 0.01, *** *p* < 0.001 compared with the control.

L-DOPA was used as a substrate for tyrosinase activity to determine the effect of morin on mushroom tyrosinase activity. Morin was added at concentrations of 25, 50, 100, 250, and 500 μM. Morin significantly increased mushroom tyrosinase activity and intracellular tyrosinase in a concentration-dependent manner (Figure 3). These results indicated that morin directly and indirectly increased tyrosinase activity. Furthermore, morin implied the possibility of regulating the expression of melanogenic enzymes.

### 2.4. Effects of Morin on Melanogenic Enzymes

**Figure 4:**
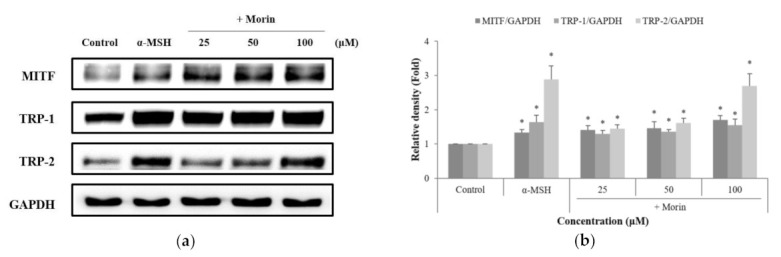
Effects of morin on the protein levels of melanogenic enzymes (TRP-1, TRP-2) and MITF. B16F10 mouse melanoma cells were treated with morin or α-MSH at the indicated concentration for 24 h. (**a**) MITF, TRP-1, and TRP-2 protein expressions were detected by western blotting. (**b**) Results were normalized against GAPDH expression. The data are presented as the mean ± SD of at least three independent experiments; * *p* < 0.05 compared with the control.

The effects of morin on melanogenic enzymes were determined using western blotting. Morin induced the protein expression of TRP-1 and TRP-2 in a dose-dependent manner. To understand TRP-1 and TRP-2 gene transcription, we examined the effect of morin on the expression of the major transcription factor MITF. The expression of MITF and melanogenesis-related proteins increased after treatment with morin (Figure 4). These results suggest that morin-induced melanin synthesis is mediated by the upregulation of melanogenic enzymes and MITF at the protein level.

### 2.5. Effects of Morin on the Phosphorylation of ERK, JNK, and p38 in the MAPK Pathway

**Figure 5:**
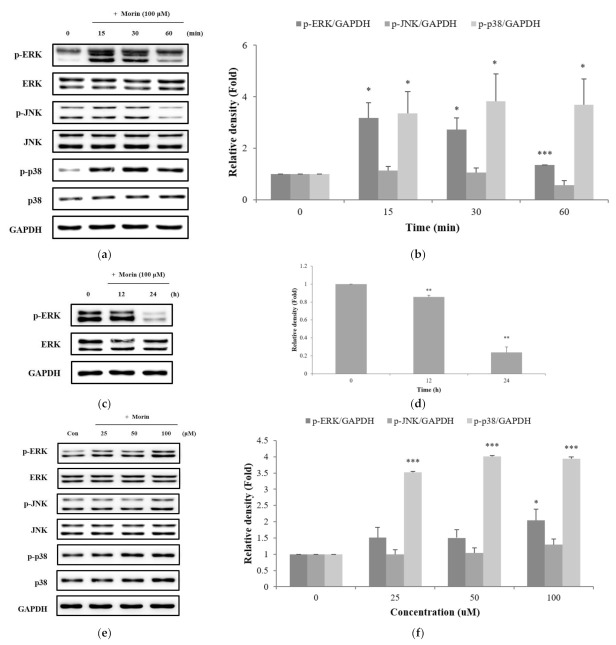
Effects of morin on the protein levels of phosphate ERK, JNK, and p38. B16F10 mouse melanoma cells were treated with morin at (**a**,**c**) various times, and (**e**) concentrations. Phosphate ERK, JNK, and p38 protein expression were detected by western blotting. (**b**,**d**,**f**) Results were normalized against GAPDH expression. The data are presented as the mean ± SD of at least three independent experiments; * *p* < 0.05, ** *p* < 0.01, *** *p* < 0.001 compared with the control.

We examined the MAPK signaling pathway to identify the molecular mechanism by which morin induces melanin production. As presented in Figure 5a,b, analysis of protein expression over time showed that phosphorylated ERK increased from 0 to 15 min and decreased after 15 min. In addition, phosphorylated ERK decreased by treatment with morin at late time points (Figure 5c,d). Phosphorylation of p38 was stimulated by morin from 0 to 30 min (Figure 5a,b). We also confirmed the expression level of MAPK by treating morin at various concentrations (25, 50, and 100 μM) for 30 min. Phosphorylated ERK and p38 significantly increased the expression levels in a concentration-dependent manner (Figure 5e,f). However, the expression level of JNK was not increased by morin.

### 2.6. Effects of Morin on MAPK Signaling by Specific Inhibitors

**Figure 6:**
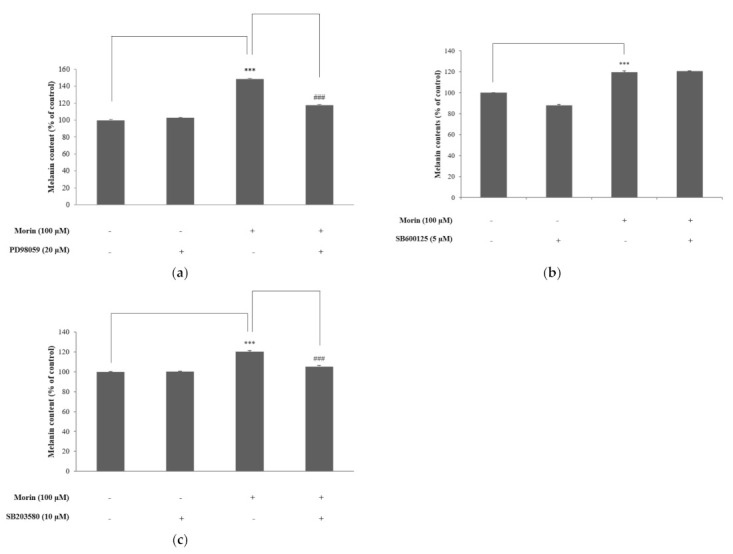
Effects of specific inhibitors of (**a**) ERK, (**b**) JNK, and (**c**) p38 on morin-induced melanin contents. Inhibitors (PD98059, SB203580) were pre-incubated with B16F10 mouse melanoma cells for 1 h before the addition of morin at 100 μM. The data are presented as the mean ± SD of at least three independent experiments; *** *p* < 0.001 compared with the control. ### *p* < 0.001 compared with morin stimulation.

To further confirm the role of the MAPK pathways in morin-induced melanin synthesis, we used the specific inhibitors ERK inhibitor (PD98059), JNK inhibitor (SP600125), and p38 inhibitor (SB203580). Cells were pretreated with specific inhibitors 1 h before the addition of morin, then incubated for 72 h for the measurement of melanin content. PD98059 (Figure 6a) and SB203580 (Figure 6c) inhibited the induction of melanin synthesis by morin. These result suggest that morin may cause melanin synthesis via ERK and p38 phosphorylation. However, the JNK inhibitor (SP600125) did not inhibit the induction of melanin synthesis by morin (Figure 6b).

## 3. Discussion

Melanin is an important determinant of human skin color that protects the skin from ultraviolet radiation and helps maintain body temperature. Additionally, the absence of melanocytes has been reported to cause skin diseases, such as vitiligo and albinism [22]. Although many melanogenesis inducers have been developed, they can cause serious side effects, such as allergies, contact dermatitis, eczema, and cytotoxicity [23]. Accordingly, many studies have been conducted on melanin biosynthesis and specific mechanisms for the development of new treatments for vitiligo [24,25].

Morin is a yellow compound that can be separated from the leaves of *Maclura pomifera* (Osage orange), *Maclura tinctoria* (Old Pustic), and *Psidium guajava* (Guava) leaves [26]. Morin has been shown to inhibit amyloid formation by islet amyloid polypeptide (amylin), decompose amyloid fibers, and inhibit IgE-mediated allergic reactions [27]. Morin treatment downregulated the expression of BLT2, NF-κB, and Th2-cytokine (IL-1β, IL-4, IL-6, IL-13, and TNF-α) in the lungs of an allergic asthma rat model [28]. However, no study on melanin biosynthesis and the molecular mechanisms of morin has been conducted.

In this study, we investigated the effect of morin on melanin biosynthesis and its molecular mechanisms to understand the signal transduction pathways in B16F10 mouse melanoma cells. Morin was not toxic up to a concentration of 100 μM, and significantly increased the concentration-dependent activity of mushroom tyrosinase activity. Morin induced melanogenesis to increase melanin biosynthesis and intracellular tyrosinase activity in B16F10 mouse melanoma cells.

Melanogenesis is directly regulated by the major enzymes TRP-1 and TRP-2 [29]. In this study, morin significantly increased the expression of TRP-1 and TRP-2. Melanogenesis enzymes are regulated by MITF, a major regulator of melanocyte development and melanogenesis [30,31]. Morin upregulated MITF expression. Considering the appearance of the protein, the isoform of MITF protein is MITF-M. The molecular weight of MITF-M in melanoma cell lines is known to be 60–65 kDa as a doublet, which represents different phosphorylation states of the protein [32]. Taken together, morin promoted melanin production by upregulating MITF and its downstream pathways, TRP-1 and TRP-2, in B16F10 mouse melanoma cells.

The mitogen-activated protein kinase (MAPK) signaling pathway is one of the major regulators of melanin biosynthesis because it is involved in the regulation of MITF expression and activation. ERK, JNK, and p38 are the major kinases mediated by several steps of activation or regulation of the MAPK pathway [33,34]. Changes in early expression of phosphorylated ERK are closely related to melanin production, and late activation of p-ERK activates phosphorylation of MITF at Ser 73, leading to ubiquitination and proteasome-mediated degradation of MITF, which is associated with the downregulation of melanogenesis [35,36,37,38]. JNK activation leads to cell differentiation, apoptosis, and melanin production in melanocytes, and p38 activation has been reported to be associated with pigmentation by increasing tyrosinase activity [39,40,41,42,43,44]. In our results, morin expression levels increased between 0 and 15 min in phosphorylated ERK and decreased after 15 min. These results indicate that the melanin synthesis effects of morin resulted from inhibition of ERK-dependent degradation of MITF. Phosphorylated p38 showed increased expression levels between 0 and 30 min. These results indicate that p38 also contributes to the melanin synthesis effect of morin. Therefore, we next confirmed the mechanism by treatment with PD98059 (ERK inhibitor), SP600125 (JNK inhibitor), and SB203580 (p38 inhibitor). As a result, ERK and p38 inhibitors inhibited morin-induced melanin synthesis. However, the JNK inhibitor did not affect melanin synthesis. It is thought that the contributions of JNK signaling is relatively weaker than ERK and p38 in the MAPK pathway.

**Figure 7:**
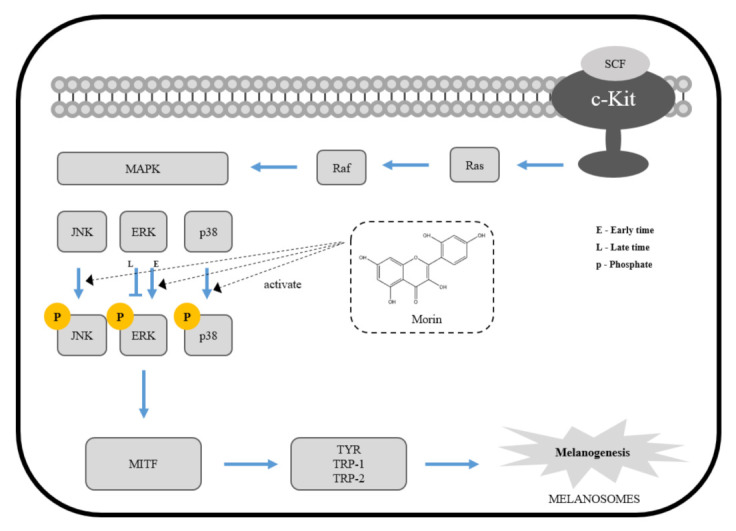
The mechanism of morin-induced melanogenesis in B16F10 mouse melanoma cells.

Taken together, our results suggest that morin enhances melanin production by upregulating MITF through the activation of ERK and p38 signaling pathways in B16F10 mouse melanoma cells (Figure 7). Several treatment methods and therapeutics, such as skin graft, lasers, vitamin D analogs, and steroids, have been used to treat vitiligo, but these methods have not been widely used because they cause long-term side effects [45,46]. From this point of view, morin is considered to be a good candidate for the treatment of vitiligo if further studies, such as efficacy and safety evaluation on humans, are successfully carried out.

## 4. Materials and Methods

### 4.1. Materials

Morin was purchased from Sigma-Aldrich (St. Louis, MO, USA). Antibodies against MITF (#12590), p-ERK (#9101), ERK (#9102), p-JNK (#4668), p-p38 (#4511), and PD98059 were purchased from Cell Signaling Technology (Beverly, MA, USA). GAPDH was purchased from Enogene (Nanjing, China). TRP-1 (sc-166857), JNK (sc-571), and p38 (H-147) were purchased from Santa Cruz Biotechnology (Santa Cruz, CA, USA). TRP-2 (ab221144) was purchased from Abcam (Cambridge, MA, USA). DMEM, fetal bovine serum (FBS), penicillin/streptomycin, and trypsin-ethylenediaminetetraacetic acid were purchased from Gibco (Grand Island, NY, USA). Dimethyl sulfoxide (DMSO), α-MSH, NaOH, 3-(4,5-dimethylthiazol-2-yl)-2,5-diphenyltetrazolium bromide (MTT), and 3,4-dihydroxy-L-phenlyanine (L-DOPA) were obtained from Sigma-Aldrich (St. Louis, MO, USA). SP600125 and SB203580 were purchased from Cayman Chemical (Ann Arbor, MI, USA). Pro-Prep lysis buffer was purchased from iNTRON Biotechnology (Seoul, Korea).

### 4.2. Cell Culture and Treatment

B16F10 melanoma cells (CRL-6475) were obtained from ATCC (American Type Culture Collection, Manassas, VA). B16F10 cells were cultured in Dulbecco’s modified Eagle’s medium (DMEM), supplemente with 10% fetal bovine serum (FBS) and 1% penicillin-streptomycin (P/S). The cells were incubated in a humidified atmosphere of 5% CO_2_ at 37 °C.

### 4.3. Measurement of Cell Viability

Cell viability was examined by the MTT assay. Briefly, B16F10 mouse melanoma cells were incubated for 24 h in the culture medium. The cells were seeded at a density of 2 × 10^4^ cells/well in 96 -well plates and incubated with various concentrations of morin (5–100 μM) for 48 h. After incubation, the cells were treated with MTT (dissolved in DMEM to 5 mg/mL) for 3 h. Following removal of the medium, 200 μL of DMSO was added to each well, and plates were gently shaken for 15 min. Absorbance was measured at 570 nm using a UV/Vis microplate reader (Thermo Fisher Scientific, Multiskan Sky, Seoul, Korea).

### 4.4. Measurement of Melanin Content

Melanin content was measured and analyzed using a previously described method with slight modifications [47]. Briefly, B16F10 mouse melanoma cells were incubated for 24 h in the culture medium. The medium was replaced with fresh medium containing morin at 25–100 μM, and α-MSH (100 nM) was used as a positive control and incubated for 72 h more. Cell pellets were dissolved in 1 N of NaOH in 10% DMSO at 80 °C for 1 h. Absorbance was measured at 475 nm using a UV/Vis microplate reader (Thermo Fisher Scientific, Multiskan Sky, Seoul, Korea).

### 4.5. Measurement of Mushroom Tyrosinase Activity

The effect of morin on mushroom tyrosinase activity was measured in a cell-free system [48]. As a substrate for tyrosinase activity, L-DOPA at a concentration of 10 mM was dissolved in sodium phosphate buffer (67 mM, pH = 6.8). After 10 min, the absorbance was measured at 475 nm using a UV/Vis microplate reader (Thermo Fisher Scientific, Multiskan Sky, Seoul, Korea).

### 4.6. Measurement of Intracellular Tyrosinase Assay

Intracellular tyrosinase activity was estimated by measuring the rate of L-DOPA oxidation as previously described, with certain modifications [49]. B16F10 melanoma cells were treated as described previously above. To measure cellular tyrosinase activity, cells were washed with cold PBS and lysed with RIPA buffer. The cell lysates were clarified by centrifugation at 32,127× *g* for 5 min. The lysates (30 μg) were dissolved in 0.1 M of sodium phosphate buffer (pH = 6.8) and treated with L-dopa (1 mg/mL) in a 96-well plate at 37 °C for 1 h. Absorbance was measured at 475 nm using a UV/Vis microplate reader (Thermo Fisher Scientific, Multiskan Sky, Seoul, Korea).

### 4.7. Western Blotting

B16F10 cells were seeded at a density of 3 × 10^5^ cells/well in a 60 mm dish and incubated with various concentrations of morin and α-MSH. Cells were washed with cold PBS and lysed in buffer for 20 min on ice. Cell lysates were centrifuged at 32,127 ×g for 5 min at 4 °C. Lysed supernatant protein was measured by the Bradford assay. The protein (10 μg) was separated with 10% sodium dodecyl sulfate polyacrylamide gel electrophoresis (SDS-PAGE) and transferred to the polyvinylidene difluoride (PVDF) membrane. The PVDF membrane was incubated for 1 h in blocking buffer (5% skim milk and 0.1% Tween 20 in TBS). The membrane was incubated with the primary antibody for 24 h at 4 °C. GAPDH was used as an internal control. After washing four times with TBS containing 0.1% Tween 20, the membrane was incubated with horseradish peroxidase-conjugated anti-mouse and anti-rabbit secondary antibodies for 1 h at room temperature. Protein band detection on the PVDF membrane was performed using Western Bright™ ECL reagent and a C300 chemiluminescence imager.

### 4.8. Statistical Analysis

The values for the independent experiments were expressed as mean ± standard deviation (mean ± SD), and statistical significance was determined using Student’s *t*-test and ANOVA. All statistical analyses were compared using the SPSS statistical software 22.0; * *p* < 0.05, ** *p* < 0.01, *** *p* < 0.001 values were considered significant differences.

## 5. Conclusions

In this study, we demonstrated that morin induced melanin synthesis and tyrosinase activity by accelerating ERK and p38 signaling pathways in B16F10 mouse melanoma cells. Taken together, these results suggest that morin may be a potential therapeutic supplement for the improvement of disorders such as hypopigmentation.

## Sample Availability

Samples of the compounds are not available from the authors.
